# Association of postoperative anaemia with prolonged hospital length of stay after upper gastrointestinal cancer surgery: a single-centre retrospective cohort study^✰^^☆^

**DOI:** 10.1016/j.bjao.2026.100648

**Published:** 2026-08-03

**Authors:** Aloysius Ng, Courtney Jones, Volker Mitteregger, Nicole Hunt

**Affiliations:** Department of Anaesthesia, Pain and Perioperative Medicine, Fiona Stanley Hospital, South Metropolitan Health Service, Murdoch, WA, Australia

**Keywords:** haemoglobin, length of stay, patient blood management, perioperative medicine, postoperative anaemia, upper gastrointestinal cancer

## Abstract

**Background:**

Prolonged hospital length of stay (LOS) after major upper gastrointestinal (GI) cancer surgery is associated with increased morbidity, mortality, and healthcare costs. Perioperative anaemia is common in this population. This study examined whether reduced haemoglobin (Hb) is independently associated with prolonged LOS after elective upper GI cancer surgery.

**Methods:**

We conducted a retrospective observational cohort study of patients undergoing elective upper GI cancer surgery at a tertiary hospital in Western Australia. Pre-, intra-, and postoperative data were obtained from a prospective institutional database. Patients were dichotomised into expected-LOS and prolonged-LOS groups based on the procedure-specific median LOS. Multivariable logistic regression was used to identify factors independently associated with prolonged LOS.

**Results:**

A total of 111 patients were included; 26 (23.4%) had prolonged LOS. These patients had lower Hb intraoperatively (106.5±17.9 *vs* 115.8±17.0 g L^−1^; *P*=0.021), on postoperative day 1 (100.1±17.3 *vs* 111.7±15.7 g L^−1^; *P*=0.002), and at first-week nadir (89.3±17.0 *vs* 104.9±16.6 g L^−1^; *P*<0.001). Reoperation (34.6% *vs* 7.1%; *P*=0.001) and unplanned ICU admission (15.4% *vs* 2.4%; *P*=0.023) were more frequent. After adjustment for these factors, a lower postoperative Hb concentration remained independently associated with a prolonged LOS. The first-week Hb nadir showed the strongest association (odds ratio 0.95 per g L^−1^; 95% confidence interval 0.92–0.98; *P*=0.002).

**Conclusions:**

Postoperative Hb decline is independently associated with prolonged LOS after elective upper GI cancer surgery. These findings support prospective evaluation of patient blood management strategies in this population.

**Editor’s key points**

• In patients undergoing major upper gastrointestinal (GI) cancer surgery, lower postoperative haemoglobin (Hb) concentrations were independently associated with prolonged hospital length of stay (LOS).
• The strongest association was observed for the Hb nadir during the first postoperative week, with each 1 g L^−1^ decrease associated with a 5% increase in the odds of prolonged hospitalisation.
• Preoperative Hb concentrations did not differ between groups, suggesting that postoperative Hb decline and recovery trajectory may be more important determinants of LOS than baseline anaemia.
• The association between postoperative Hb and prolonged LOS persisted after adjustment for reoperation and unplanned ICU admission, supporting postoperative anaemia as an independent marker of recovery.
• These findings support further prospective evaluation of perioperative patient blood management strategies, including postoperative iron replacement and Hb optimisation, in upper GI cancer surgery patients.

Upper gastrointestinal (GI) cancers impose a substantial global health burden, with persistently poor prognosis across all subtypes. Notably, 5-yr relative survival rates are among the lowest of all cancer types: 9.8% for pancreatic, 18.5% for hepatic, 20.1% for biliary, 22% for oesophageal, and 30.3% for gastric cancers.^1^^,^^2^ The patient population is typically older, functionally impaired, and comorbid, with late-stage presentation limiting resectability to 15%–50% at diagnosis.^2^ Therefore, optimising perioperative outcomes in patients who do proceed to surgery demands a coordinated multidisciplinary approach, encompassing prehabilitation, nutritional optimisation, neoadjuvant therapy, and evidence-based enhanced recovery protocols.^3^, ^4^, ^5^

Perioperative anaemia is a prevalent and clinically important comorbidity in this population. Approximately one-third of patients presenting for major elective surgery are anaemic preoperatively, and this proportion is higher still among patients with malignancy, in whom anaemia arises from multiple concurrent mechanisms: nutritional deficiencies, chronic inflammation-driven hepcidin-mediated iron sequestration, haemolysis, surgical blood loss, and chemotherapy- or radiation-induced myelosuppression.^6^, ^7^, ^8^, ^9^ Preoperative anaemia is an established independent risk factor for perioperative blood transfusion, postoperative complications, and mortality after major surgery, findings that have been confirmed in large meta-analyses and cohort studies.^10^, ^11^, ^12^, ^13^

Despite this established relationship, the specific and independent contribution of postoperative haemoglobin (Hb) decline to prolonged hospital length of stay (LOS) has not been clearly delineated in patients undergoing major upper GI cancer surgery. This distinction is clinically important. Unlike preoperative anaemia, which may prompt preoperative iron supplementation or erythropoiesis-stimulating agents, postoperative anaemia arises from a combination of surgical blood loss, haemodilution, impaired erythropoiesis driven by postoperative inflammation, and ongoing diagnostic phlebotomy.^14^^,^^15^ These mechanisms are potentially addressable through targeted postoperative patient blood management (PBM) strategies.^16^, ^17^, ^18^

Prolonged hospital LOS is not a trivial outcome. It is independently associated with hospital-acquired complications, mortality, readmission, and substantial economic burden.^19^^,^^20^ Three recent studies, Myles and colleagues^21^ (Restrictive *versus* Liberal Fluid Therapy in Major Abdominal Surgery [RELIEF] trial reanalysis), Warner and colleagues,^22^ and the international Post-operative Variability in Anaemia Treatment and Transfusion (POSTVenTT) collaborative,^23^ have each demonstrated that postoperative anaemia is independently associated with adverse outcomes including prolonged LOS, 30-day readmission, impaired quality of recovery, and disability-free survival after major abdominal surgery. However, none of these studies focused specifically on upper GI cancer surgery, a cohort uniquely characterised by extensive visceral resections, prolonged operative times, neoadjuvant treatment effects, and high baseline rates of nutritional compromise.

Therefore, we hypothesised that modifiable postoperative factors, particularly Hb decline, could contribute to prolonged hospital LOS independent of preoperative and intraoperative variables in this high-risk population. We conducted a retrospective cohort study evaluating pre-, intra-, and postoperative factors associated with prolonged hospital LOS in patients undergoing elective upper GI cancer surgery at a major Australian tertiary referral centre.

## Methods

### Study design and population

This study constitutes a complete consecutive institutional cohort over a predefined 12-month period. We performed a retrospective observational cohort study of all patients who underwent elective upper GI cancer surgery at Fiona Stanley Hospital, Perth, Western Australia, between January 1, 2021, and December 31, 2021. The inclusion criteria were all adult patients undergoing elective major resection upper GI surgery identified by operation codes (pancreaticoduodenectomy [Whipple’s procedure], oesophagectomy, hepatectomy, distal pancreatectomy with splenectomy, and gastrectomy) from the Theatre Management System (TMS) database. These procedures are performed by a dedicated hepatopancreatobiliary and upper GI surgical team at a single high-volume tertiary referral centre and encompass the spectrum of major resection surgery for upper GI malignancy in our institution. The exclusion criteria were operations with final histopathology demonstrating non-malignant (benign) disease, emergency (unplanned) procedures, and non-resection or palliative operations.

A total of 120 patients were initially screened from TMS operation codes. Nine patients were excluded after histopathological review (final pathology demonstrating benign disease), yielding a final analytical cohort of 111 patients.

### Data collection

Pre-, intra-, and postoperative data were extracted from a prospective institutional database supplemented by systematic retrospective review of electronic medical and anaesthetic records. Preoperative functional capacity was assessed using the Duke activity status index (DASI), a validated 12-item questionnaire that estimates metabolic equivalents of cardiac reserve, and frailty was quantified using the Edmonton frailty scale (EFS), a validated 10-item multidimensional frailty assessment tool.^24^^,^^25^ Hb concentrations were recorded at the following time points: preoperative, lowest intraoperative, lowest day 1 postoperative, nadir days 1–7, and nadir days 8–30. All Hb values are reported in g L^−1^.

### Outcome definition and LOS categorisation

The primary outcome was prolonged hospital LOS, defined as exceeding the procedure-specific median LOS for that cohort. Procedure-specific medians were as follows: pancreaticoduodenectomy and oesophagectomy, 10 days; gastrectomy, 9 days; distal pancreatectomy and splenectomy, 8 days; and hepatectomy, 5 days.^26^ Patients were accordingly dichotomised into expected-LOS and prolonged-LOS groups. This procedure-specific approach accounts for inherent differences in expected postoperative recovery trajectories across major upper GI procedures.

### Statistical analysis

Statistical analyses were performed using IBM SPSS Statistics version 26. Continuous variables are presented as mean±standard deviation. Normality was assessed using the Shapiro–Wilk test. Between-group comparisons used the independent-samples *t*-test or Mann–Whitney *U*-test for continuous variables and the χ^2^ or Fisher’s exact test for categorical variables. Variables significant at P<0.05 on univariate logistic regression (unplanned ICU admission, reoperation, postoperative transfusion, and all Hb time-point variables) were considered for multivariable modelling. Because serial postoperative Hb measurements are highly correlated, each Hb time point was assessed in a separate multivariable model rather than jointly, to avoid collinearity; reoperation and unplanned ICU admission were retained in every model as they were the only non-Hb variables significant on univariate analysis. Neoadjuvant radiation therapy timing, although significant in the group-level comparison (Table 2), was not significant in univariate logistic regression for prolonged LOS and was therefore not entered into the multivariable models; its potential confounding role is addressed in the Discussion. Postoperative transfusion, despite univariate significance, was excluded from the primary multivariable models given its expected collinearity with Hb concentration (transfusion trigger); a sensitivity analysis re-entering transfusion alongside each Hb variable, reoperation, and unplanned ICU admission is reported alongside the primary results.

## Results

### Patient characteristics

Notably, 111 patients underwent elective upper GI cancer surgery during the study period. The mean age was 64.0 yr, 71 (64.0%) were male, and most had ASA physical status 3 (expected-LOS group 70.6%; prolonged-LOS group 65.4%). There were no statistically significant differences between groups in age, sex, ASA status, BMI, DASI score, EFS score, or smoking and alcohol use history (Table 1).

**Table 1:** Patient characteristics. DASI, Duke activity status index; LOS, length of stay; sd, standard deviation. ∗Independent-samples *t*-test. ^†^χ^2^ test. ^‡^Mann–Whitney *U*-test.

	Expected LOS (*n*=85)	Prolonged LOS (*n*=26)	*P*-value
Age (yr), mean (sd)	64.0 (10.8)	64.0 (14.0)	0.988∗
Male sex, *n* (%)	57 (67.1)	14 (53.8)	0.219^†^
ASA physical status, *n* (%)			0.186^†^
1	–	1 (3.8)	
2	25 (29.4)	8 (30.8)	
3	60 (70.6)	17 (65.4)	
BMI (kg/m^2^), mean (sd)	26.8 (5.4)	26.4 (4.7)	0.862^‡^
DASI score, mean (sd)	39.8 (14.1)	32.5 (15.2)	0.083^‡^
Edmonton frailty scale score, mean (sd)	3.70 (2.7)	5.4 (4.2)	0.141^‡^
Smoking status, *n* (%)			0.723^†^
Non-smoker	35 (41.2)	12 (46.2)	
Ex-heavy smoker	10 (11.8)	4 (15.4)	
Light smoker	40 (47.1)	10 (38.5)	
Alcohol use, *n* (%)			0.400^†^
None	35 (41.2)	8 (30.8)	
Ex-heavy	6 (7.1)	2 (7.7)	
Light	41 (48.2)	13 (50.0)	
Heavy	3 (3.5)	3 (11.5)	

### Perioperative and intraoperative factors

Twenty-six patients (23.4%) experienced prolonged hospital LOS. Importantly, preoperative Hb concentrations and rates of preoperative iron deficiency were comparable between groups (132.1±12.9 g L^−1^ *vs* 130.8±12.7 g L^−1^; *P*=0.649, and 23.5% *vs* 15.4%; *P*=0.300, respectively), indicating that baseline anaemia did not differentiate the groups. Neoadjuvant radiation therapy timing differed significantly between groups (*P*=0.016), with the expected-LOS group more likely to have received radiation 6–12 weeks before surgery. No significant differences were observed in neoadjuvant chemotherapy timing, timing of preoperative clinic review, or treatment of iron deficiency (Table 2). Surgical procedure distribution, approach, operative duration, and estimated blood loss were similar between groups, although estimated blood loss trended higher in the prolonged-LOS group (818.9±969.2 *vs* 494.7±591.7 ml; *P*=0.085) (Table 3).

**Table 2:** Perioperative factors. LOS, length of stay. ∗χ^2^ test. ^†^Statistically significant. ^‡^Fisher’s exact test.

	Expected LOS (*n*=85)	Prolonged LOS (*n*=26)	*P*-value
Neoadjuvant chemotherapy timing, *n* (%)			0.374∗
None	50 (58.8)	19 (73.1)	
<6 weeks	4 (4.7)	2 (7.7)	
6–12 weeks	28 (32.9)	4 (15.4)	
>12 weeks	3 (3.5)	1 (3.8)	
Neoadjuvant radiation therapy timing, *n* (%)			0.016∗^,†^
None	70 (82.4)	22 (84.6)	
6–12 weeks	14 (16.5)	1 (3.8)	
>12 weeks	1 (1.2)	3 (11.5)	
Timing of preoperative clinic review, *n* (%)			0.186∗
<1 week	21 (24.7)	12 (46.2)	
<2 weeks	20 (23.5)	3 (11.5)	
<4 weeks	22 (25.9)	5 (19.2)	
>4 weeks	21 (24.7)	6 (23.1)	
Preoperative iron deficiency, *n* (%)	20 (23.5)	4 (15.4)	0.300^‡^
Treatment of iron deficiency, *n* (%)			0.307∗
None	63 (74.1)	16 (61.5)	
Preoperative replacement	2 (2.4)	2 (7.7)	
Intraoperative replacement	5 (5.9)	1 (3.8)	
Postoperative replacement	13 (15.3)	7 (26.9)

**Table 3:** Intraoperative factors. LOS, length of stay; sd, standard deviation. ∗χ^2^ test. ^†^Independent-samples *t*-test.

	Expected LOS (*n*=85)	Prolonged LOS (*n*=26)	*P*-value
Surgical procedure, *n* (%)			0.155∗
Pancreaticoduodenectomy (Whipple’s)	33 (38.8)	8 (30.8)	
Oesophagectomy	18 (21.2)	2 (7.7)	
Hepatectomy	13 (15.3)	9 (34.6)	
Distal pancreatectomy and splenectomy	6 (7.1)	3 (11.5)	
Gastrectomy	15 (17.6)	4 (15.4)	
Surgical approach, *n* (%)			0.152∗
Laparoscopic	47 (55.3)	10 (38.5)	
Laparoscopic-assisted open	4 (4.7)	1 (3.8)	
Open	34 (40.0)	14 (53.8)	
Robotic-assisted laparoscopic	–	1 (3.8)	
Operative duration (h), mean (sd)	7.0 (2.2)	7.2 (2.4)	0.824^†^
Estimated blood loss (ml), mean (sd)	494.7 (591.7)	818.9 (969.2)	0.085^†^

### Postoperative factors

Significant postoperative differences were identified between groups. Unplanned ICU/high dependency unit admission occurred more frequently in the prolonged-LOS group (15.4% *vs* 2.4%; *P*=0.023), as did reoperation (34.6% *vs* 7.1%; *P*=0.001) and prolonged ICU stay (3–6 days: 42.3% *vs* 21.2%; *P*=0.012). Hospital readmission rates did not differ significantly between groups (15.3% *vs* 15.4%; *P*=0.630) (Table 4).

**Table 4:** Postoperative factors. HDU, high dependency unit; LOS, length of stay. ∗Statistically significant. ^†^Fisher’s exact test. ^‡^χ^2^ test.

	Expected LOS (*n*=85)	Prolonged LOS (*n*=26)	*P*-value
Unplanned ICU/HDU admission, *n* (%)	2 (2.4)	4 (15.4)	0.023∗^,†^
Reoperation, *n* (%)	6 (7.1)	9 (34.6)	0.001∗^,‡^
Readmission to hospital, *n* (%)	13 (15.3)	4 (15.4)	0.630^†^
ICU length of stay, *n* (%)			0.012∗^,‡^
Ward only (no ICU)	1 (1.2)	2 (7.7)	
Within 48 h	45 (52.9)	8 (30.8)	
3–6 days	18 (21.2)	11 (42.3)	
>1 week	–	1 (3.8)	
Mortality, *n* (%)			0.305^‡^
Alive	75 (88.2)	25 (96.2)	
Death <3 months	1 (1.2)	–	
Death <12 months	1 (1.2)	1 (3.8)	
Death <2 yr	8 (9.4)	–	

### Haemoglobin and transfusion

Preoperative Hb concentrations were comparable between groups (132.1±12.9 g L^−1^ *vs* 130.8±12.7 g L^−1^; *P*=0.649). Patients with prolonged LOS had significantly lower Hb at all subsequent time points: lowest intraoperative Hb (106.5±17.9 g L^−1^ *vs* 115.8±17.0 g L^−1^; *P*=0.021), lowest Hb on day 1 after surgery (100.1±17.3 g L^−1^ *vs* 111.7±15.7 g L^−1^; *P*=0.002), first-week Hb nadir (89.3±17.0 g L^−1^ *vs* 104.9±16.6 g L^−1^; *P*<0.001), and Hb nadir during days 8–30 (95.5±14.2 g L^−1^ *vs* 108.4±17.0 g L^−1^; *P*=0.003). Postoperative blood transfusion was more frequent in the prolonged-LOS group (23.1% *vs* 5.9%; *P*=0.019) (Table 5).

**Table 5:** Hb and transfusion data. Hb, haemoglobin; LOS, length of stay; sd, standard deviation. ∗Independent-samples *t*-test. ^†^Statistically significant. ^‡^Mann–Whitney *U*-test. ^¶^Fisher’s exact test.

Hb measure (g L^−1^), mean (sd)	Expected LOS (*n*=85)	Prolonged LOS (*n*=26)	*P*-value
Preoperative Hb (g L^−1^)	132.1 (12.9)	130.8 (12.7)	0.649∗
Lowest intraoperative Hb (g L^−1^)	115.8 (17.0)	106.5 (17.9)	0.021∗^,†^
Lowest Hb day 1 postoperative (g L^−1^)	111.7 (15.7)	100.1 (17.3)	0.002^†,‡^
Hb nadir days 1–7 (g L^−1^)	104.9 (16.6)	89.3 (17.0)	<0.001∗^,†^
Hb nadir days 8–30 (g L^−1^)	108.4 (17.0)	95.5 (14.2)	0.003∗^,†^
Intraoperative transfusion, *n* (%)	3 (3.5)	1 (3.8)	0.662^¶^
Postoperative transfusion, *n* (%)	5 (5.9)	6 (23.1)	0.019^†,¶^

### Regression analysis

On univariate logistic regression, variables significantly associated with prolonged hospital LOS included unplanned ICU admission (OR 7.91; 95% CI 1.36–46.11; *P*=0.022), reoperation (OR 6.88; 95% CI 2.16–21.92; *P*=0.001), postoperative transfusion (OR 4.80; 95% CI 1.33–17.33; *P*=0.017), and all Hb time-point variables. On multivariate analysis adjusting for unplanned ICU admission and reoperation, lower Hb at all postoperative time points remained independently associated with prolonged LOS. The first-week Hb nadir was the most strongly associated Hb variable (OR 0.95; 95% CI 0.92–0.98; *P*=0.002) (Table 6).

**Table 6:** Univariable and multivariable binary logistic regression analysis for predictors of prolonged hospital length of stay. Each postoperative Hb time point was assessed in a separate multivariable model adjusted for reoperation and unplanned ICU admission (the only non-Hb covariates significant on univariable analysis); models were not combined because of collinearity among serial Hb measures. Reoperation and unplanned ICU admission coefficients are reported separately for each model, as they varied according to the complete-case sample available for that Hb time point (n=67–109), reflecting differential missingness across postoperative Hb measures (see Limitations). Neoadjuvant radiation therapy timing and postoperative transfusion, though significant on relevant comparisons, were not retained in these models (see Methods and Discussion for rationale and sensitivity analysis). CI, confidence interval; Hb, haemoglobin; NS, not significant; OR, odds ratio.

Model / Variable	OR	95% CI	P-value
***Univariable analysis (all variables, single cohort n=110–111)***			
Neoadjuvant radiation therapy	NS	—	—
Unplanned ICU admission	7.91	1.36–46.11	0.022
Reoperation	6.88	2.16–21.92	0.001
ICU length of stay	NS	—	—
Lowest intraoperative Hb (g L^-1^)	0.97	0.94–1.00	0.026
Lowest Hb day 1 postoperative (g L^-1^)	0.96	0.93–0.99	0.003
Hb nadir days 1–7 (g L^-1^)	0.94	0.92–0.97	<0.001
Hb nadir days 8–30 (g L^-1^)	0.95	0.92–0.99	0.005
Postoperative transfusion	4.80	1.33–17.33	0.017
***Model 1: adjusted for lowest intraoperative Hb (n=105)***			
Lowest intraoperative Hb (g L^-1^)	0.96	0.93–0.99	0.018
Reoperation	8.48	2.39–30.00	0.001
Unplanned ICU admission	6.80	1.03–45.00	0.047
***Model 2: adjusted for lowest Hb day 1 postoperative (n=109)***			
Lowest Hb day 1 postoperative (g L^-1^)	0.95	0.92–0.98	0.003
Reoperation	9.39	2.51–35.09	0.001
Unplanned ICU admission	4.80	0.64–35.99	0.127
***Model 3: adjusted for Hb nadir days 1–7 (n=109)***			
Hb nadir days 1–7 (g L^-1^)	0.95	0.92–0.98	0.002
Reoperation	8.43	2.09–34.06	0.003
Unplanned ICU admission	4.00	0.46–34.63	0.208
***Model 4: adjusted for Hb nadir days 8–30 (n=67)***			
Hb nadir days 8–30 (g L^-1^)	0.95	0.92–0.99	0.014
Reoperation	3.32	0.89–12.35	0.073
Unplanned ICU admission	8.82	0.57–137.01	0.120

## Discussion

This retrospective cohort study demonstrates that postoperative Hb decline, quantified as the change in Hb concentration across multiple time points from the intraoperative period through the first month after surgery, is independently associated with prolonged hospital LOS after elective upper GI cancer surgery. Critically, this association persisted after multivariate adjustment for reoperation and unplanned ICU admission, two strong markers of postoperative clinical deterioration. The first-week Hb nadir was the most strongly associated Hb variable, with a 5% increase in odds of prolonged LOS for each 1 g L^−1^ decrement. Patients with prolonged LOS had a mean first-week nadir of 89.3 g L^−1^ compared with 104.9 g L^−1^ in those with expected LOS.

These findings add to a growing body of evidence linking postoperative anaemia to adverse surgical outcomes. The landmark reanalysis by Myles and colleagues^21^ of the RELIEF trial encompassing 2983 patients across 47 centres demonstrated that postoperative anaemia was independently associated with persistent disability or death at 90 days, impaired quality of recovery, longer hospital stay, and increased hospital readmissions after major abdominal surgery.^21^ Warner and colleagues,^22^ in a two-cohort study of more than 32 700 surgical patients, found that each 10 g L^−1^ decrement in discharge Hb was associated with an 8%–9% increase in the hazard for 30-day readmission. The international POSTVenTT prospective cohort study,^23^ involving 101 hospitals across 13 countries, confirmed that postoperative anaemia at discharge independently increased unplanned readmission and the risk of functional deterioration. Our findings are consistent with and extend these observations to the specific context of upper GI cancer surgery, where the consequences of anaemia may be compounded by disease biology and treatment effects.

A critical distinguishing feature of our data is that preoperative Hb concentrations were not statistically different between groups (132.1 g L^−1^ *vs* 130.8 g L^−1^; *P*=0.649). This confirms that it is the postoperative Hb trajectory, and not the baseline, that drives prolonged LOS in this cohort. This finding is consistent with the emerging clinical distinction between preoperative and postoperative anaemia as distinct entities with different mechanisms, timelines, and therapeutic implications.^14^^,^^15^^,^^27^ Postoperative anaemia arises from surgical blood loss, haemodilution, impaired erythropoiesis driven by postoperative inflammation and elevated hepcidin, and ongoing diagnostic phlebotomy.^14^^,^^15^ Notably, the anaemia persisted and, in some patients, deepened over the first month after surgery (Hb nadir days 8–30: 95.5 g L^−1^ in the prolonged-LOS group), suggesting inadequate Hb recovery during the inpatient period rather than a transient acute-phase response.

The mechanisms by which postoperative anaemia may prolong hospital LOS are multifactorial. At a physiological level, reduced Hb impairs oxygen delivery to tissues, potentially delaying wound healing, increasing susceptibility to infection, impairing cardiorespiratory functional reserve during mobilisation, and reducing capacity for early rehabilitation.^11^^,^^12^ In the context of upper GI cancer surgery specifically, patients frequently require nutritional support, physiotherapy, stoma or drain management, and multidisciplinary team input during their inpatient recovery, all of which may be compromised by anaemia-related fatigue and reduced functional capacity. Anaemia has also been associated with postoperative delirium, a complication that independently prolongs hospital LOS and impairs cognitive recovery.^28^ In patients with malignancy, postoperative anaemia may additionally delay adjuvant chemotherapy or radiation commencement, compounding longer-term oncological outcomes.^29^

The risks associated with blood transfusion as a corrective strategy in this population warrant careful consideration. Transfusion-related immune modulation (TRIM) has been proposed as a mechanism by which allogeneic blood transfusion may suppress recipient immune surveillance, theoretically increasing the risk of postoperative infection, cancer recurrence, and metastasis.^30^^,^^31^ The molecular mechanisms of TRIM and its clinical significance in critically ill and oncological patients have been recently reviewed.^31^^,^^32^ It should be noted that the application of TRIM to our cohort remains speculative, as we did not measure immunological endpoints and any causal inference would exceed the scope of this retrospective analysis.

Although evidence in gastric and oesophageal cancer remains mixed, several large studies have not demonstrated a significant independent effect on survival; a cautious and evidence-based approach to transfusion thresholds is prudent.^33^, ^34^, ^35^, ^36^ The international consensus statements on perioperative anaemia management and the International Consensus Conference on Anemia Management in Surgical Patients guidelines recommend restrictive transfusion strategies in stable patients (Hb <70 to 80 g L^−1^), with proactive treatment of the underlying causes of anaemia rather than reactive transfusion as the preferred approach.^16^^,^^17^^,^^37^

From a clinical practice perspective, our findings support integrating structured PBM programmes into perioperative care pathways for major upper GI cancer surgery. PBM is a multidisciplinary, evidence-based approach aimed at optimising Hb, minimising blood loss, and rationalising transfusion, with the overarching goal of improving patient outcomes.^46^ In the preoperative phase, screening for and treating iron deficiency and anaemia with i.v. iron or erythropoiesis-stimulating agents in appropriately selected patients represents a first-line strategy.^16^^,^^17^ The PREVENTT trial demonstrated that preoperative i.v. iron resulted in higher Hb at 6 months and fewer 8-week hospital readmissions, despite not reducing the primary endpoints of transfusion or death.^39^ In the postoperative phase, targeted strategies to minimise Hb decline include restrictive diagnostic phlebotomy volumes, cell salvage where applicable, judicious use of intraoperative haemostatic agents including tranexamic acid, and prompt identification and treatment of postoperative iron deficiency with i.v. iron.^16^^,^^38^ The emerging evidence that postoperative i.v. iron supplementation can accelerate Hb recovery and may reduce readmissions provides a potential future therapeutic target in this population.^40^

These findings have particular clinical significance in the upper GI oncological surgery population. Unlike many other surgical cohorts, patients undergoing major upper GI cancer surgery are frequently affected by disease-related nutritional compromise, neoadjuvant therapy-induced myelosuppression, and impaired GI iron absorption after resection. These factors may both deepen postoperative Hb nadirs and impair erythropoietic recovery, making this population uniquely vulnerable to the downstream functional consequences of anaemia. This raises the question of whether current transfusion and Hb thresholds are appropriately calibrated for this specific surgical setting, in which the physiological demands of early rehabilitation, nutritional restoration, and wound healing may warrant more proactive Hb management than the conventional restrictive strategy allows. The emerging evidence on iron deficiency without anaemia as a clinically meaningful entity in this population is also relevant: iron depletion impairs oxidative metabolism and cellular energy production even in the absence of anaemia, and its prevalence among patients with perioperative upper GI cancer is likely underappreciated.

Preoperative identification and treatment of iron deficiency even when Hb remains above anaemia thresholds may enhance erythropoietic reserve for the perioperative period and accelerate Hb recovery. In the postoperative phase, early i.v. iron supplementation is a pragmatic, evidence-informed intervention to facilitate Hb recovery, reduce readmission risk, and potentially support earlier functional rehabilitation. Prospective evaluation of targeted iron repletion protocols specifically in upper GI cancer surgery cohorts is warranted.

Preoperative functional status showed a trend towards worse scores in the prolonged-LOS group: DASI 32.5 *vs* 39.8 (*P*=0.083) and EFS score 5.4 *vs* 3.7 (*P*=0.141). Although neither reached statistical significance in this sample, these trends are consistent with published data demonstrating that lower preoperative functional capacity and greater frailty are associated with higher rates of postoperative complications and prolonged LOS.^24^^,^^41^

The heterogeneity of the surgical cohort is notable, as hepatectomy and pancreaticoduodenectomy are associated with higher baseline complication rates and ICU utilisation than oesophagectomy or gastrectomy. In our institution, these procedures are performed within a unified hepatopancreatobiliary and upper GI surgical programme under the same multidisciplinary team and are included in established upper GI cancer registries.^2^ We applied procedure-specific LOS thresholds to account for differences in expected recovery trajectories, which mitigates but does not eliminate the effect of procedural heterogeneity. The trend towards higher estimated blood loss in the prolonged-LOS group (818.9 *vs* 494.7 ml; *P*=0.085) may reflect greater procedural complexity or an underlying disease stage and may independently contribute to postoperative Hb decline.

This study has several limitations. The single-centre retrospective design limits generalisability and introduces the potential for unmeasured confounding. The sample size of 111 patients limits statistical power, particularly for subgroup analyses. We did not apply a WHO-based binary anaemia threshold; instead, we treated serial Hb concentrations as a continuous variable across multiple time points, which we consider a methodological strength, as it captures the dynamic postoperative Hb trajectory and avoids sensitivity to a single threshold.^45^ The WHO anaemia thresholds (Hb <130 g L^−1^ in males, <120 g L^−1^ in females) may have limited utility in this context, as most of this cohort fall below these values at multiple perioperative time points owing to surgical blood loss and possible haemodilution; therefore, continuous Hb modelling provides greater discriminative ability.

The procedure-specific LOS thresholds were derived from published benchmark medians rather than from within-cohort interquartile range (IQR), given the small sample size and heterogeneous procedure mix; future studies using institution-specific IQR-based thresholds would be informative.^26^ Postoperative transfusion was significantly associated with prolonged LOS on univariate analysis (OR 4.80) but was not retained in the final multivariate model. This co-linearity with Hb concentration reflects the expected relationship between anaemia severity and transfusion trigger, and therefore, the independent association with Hb encompasses some of this effect. Hb nadir during days 8-30 was not available for all patients: 39 of 85 expected-LOS patients (45.9%) and 3 of 26 prolonged-LOS patients (11.5%) lacked a measurement in this window, most plausibly because patients with an uncomplicated, shorter admission were discharged before day 8. This missingness is therefore unlikely to be random with respect to LOS group, and the corresponding multivariable model (n=67) is fitted on a complete-case sample skewed towards more complicated postoperative courses. The adjustor coefficients in this model were unstable (reoperation OR 3.32, 95% CI 0.89-12.35, P=0.073; unplanned ICU admission OR 8.82, 95% CI 0.57-137.01, P=0.120). The Hb nadir days 8-30 association itself (OR 0.95, 95% CI 0.92-0.99) should be interpreted with more caution than earlier time points; this differential missingness is acknowledged as a limitation.

To assess whether the association between Hb and prolonged LOS was driven by low Hb itself or by transfusion as an intermediate, we performed a sensitivity analysis that included postoperative transfusion as a covariate alongside Hb, reoperation, and unplanned ICU admission in the multivariable model. In this model, the association between first-week Hb nadir and prolonged LOS remained statistically significant (OR 0.96; 95% CI 0.92–0.99; *P*=0.012), whereas transfusion was not independently retained. This supports the conclusion that Hb concentration, rather than transfusion, is the primary variable associated with prolonged LOS in this cohort, although transfusion itself may contribute to prolonged LOS through independent mechanisms including TRIM and logistic factors.

Formal Clavien-Dindo complication grading was not systematically recorded in our prospective database during data collection, precluding complication-stratified analyses. Future studies should incorporate formal Clavien-Dindo complication grading to more rigorously characterise the relationship among postoperative complications, anaemia, and LOS and should consider stratified analyses by procedure type and complexity.^44^

We observed that cumulative 2-year mortality was numerically higher in the expected-LOS group than the prolonged-LOS group (11.8% [10/85] vs 3.8% [1/26]), a counter-intuitive finding that may reflect survival bias (patients dying early or experiencing the most severe complications remaining hospitalised longer and thus being classified as prolonged LOS, although those discharged at expected intervals had sufficient functional reserve to survive to 2 yr), small sample size, or unmeasured oncological confounders; this finding warrants further investigation in larger prospective cohorts. Neoadjuvant radiation timing differed significantly between groups and may act as a confounder through myelosuppressive effects on Hb recovery. Although this was captured as a covariate in the univariate analysis, it did not reach significance and was not retained in the multivariate model; its potential confounding role is acknowledged. Despite these limitations, the study is strengthened by its prospective database, comprehensive perioperative data collection, analysis of serial Hb time points, and use of multivariate regression to identify factors independently associated with prolonged LOS.

In conclusion, lower postoperative Hb concentration is independently associated with prolonged hospital LOS after elective upper GI cancer surgery, and this association persists after adjustment for reoperation and unplanned ICU admission. The postoperative Hb trajectory seems to be a key modifiable factor contributing to prolonged hospital LOS. These findings have direct implications for perioperative care pathways and support further prospective evaluation of PBM strategies in patients undergoing major upper GI cancer surgery. Prospective trials are needed to determine whether targeted perioperative anaemia management — including preoperative iron optimisation, intraoperative blood conservation, and postoperative iron supplementation — can reduce hospital LOS and improve short- and long-term outcomes including quality of life, cancer recurrence, and survival in this population.

## Authors’ contributions

Study design: AN, VM

Data collection and interpretation: NH, CJ

Data interpretation: VM

Data management and interpretation: AN

Literature search: AN

Statistical analyses: AN

Manuscript drafting: AN

Manuscript review: VM, NH, CJ

Ethics approval: NH, CJ

Database design: NH, CJ

## Data availability

Clinical data are stored in local databases within the South Metropolitan Health System in accordance with the Health Services Act 2016. Datasets are available from the corresponding author on reasonable request.

## Ethics and governance

South Metropolitan Health Service (SMHS) site-based governance approval was obtained (GEKO 47285; approved for publication June 19, 2023). The project was assessed as a quality improvement activity under the SMHS Governance, Evidence, Knowledge and Outcomes system and therefore did not require review or approval by the Human Research Ethics Committee. Individual patient consent was waived given the retrospective observational design. This study was conducted and reported in accordance with the Strengthening the Reporting of Observational Studies in Epidemiology statement.^46^

## Declaration of generative AI and AI-assisted technologies in the writing process

During the preparation of this work, the authors used Gemini and Perplexity to review new literature that has been published. After using this tool/service, the authors reviewed and edited the content as needed and take full responsibility for the content of the published article.

## Declarations of interest

The authors declare that they have no conflicts of interest.
